# Transcriptomic dataset of *Phaseolus vulgaris* leaves in response to the inoculation of pathogenic *Xanthomonas citri* pv. *fuscans* and its type III secretion system-defective mutant *hrcV*

**DOI:** 10.1016/j.dib.2024.110938

**Published:** 2024-09-21

**Authors:** Christopher Gihaut, Chrystelle Brin, Martial Briand, Jérôme Verdier, Matthieu Barret, Thomas Roitsch, Tristan Boureau

**Affiliations:** a INRAE, IRHS, SFR QUASAV, Institut Agro, Université Angers, Angers F-49000, France; b Department of Plant and Environmental Sciences, Copenhagen Plant Science Centre, University of Copenhagen, Taastrup, Denmark

**Keywords:** Common bean, Plant defence, Type III effectors, ETS, PTI, RNAseq

## Abstract

*Xanthomonas citri* pv. *fuscans* (*Xcf*) and *Xanthomonas phaseoli* pv. *phaseoli* (*Xpp*) are responsible for the Common Bacterial Blight (CBB), a major common bean (*Phaseolus vulgaris*) disease. The pathogenicity of *Xcf* and *Xpp* is known to be dependent upon a functional Type III Secretion System (T3SS) allowing the injection of numerous bacterial Type III Effectors (T3Es) into plant cells. T3Es have been described as able to disrupt plant defence and manipulate plant metabolism.

In this work we described the transcriptomic response of one susceptible (Flavert) and one resistant (Vezer) cultivars of *P. vulgaris* to the inoculation of the virulent strain *Xcf* CFBP4885 or its avirulent T3SS-defective *hrcV* mutant (CFBP13802).

Leaves of both bean cultivars were infiltrated with water or bacterial suspensions. Inoculated leaves were sampled at 24 or 48 h post inoculation (hpi). The experiment was independently repeated three times for total RNA extraction and sequencing analysis. Library construction and total RNA sequencing were performed with BGISEQ-500 at Beijing Genomics Institute (BGI, Hong-Kong), generating an average of 24M of paired-end reads of 100bp per sample. FastQC was used to check reads quality. Mapping analyses were made using a quasi-mapping alignment from Salmon (version 1.2.1) against the *Phaseolus vulgaris* reference genome (version 2.1), revealing the expression profiles of 36,978 transcripts in leaf tissues.

Fastq raw data and count files from 36 samples are available in the Gene Expression Omnibus (GEO) repository of the National Center for Biotechnology Information (NCBI) under the accession number GSE271236.

This dataset is a valuable resource to investigate the role of T3Es in subverting the cellular functions of bean.

Specifications Table

**Table:** 

Subject	*Agricultural and Biological Sciences*
Specific subject area	Omics: Transcriptomics Plant Science: Plant-Microbe Interaction
Type of data	Filtered raw reads (fastq), total counts table (count.txt), and total normalised count table (TPM.txt)
Data collection	Leaves from susceptible and resistant *Phaseolus vulgaris* cultivars were inoculated with the virulent *Xcf* strain CFBP4885 (wt) or its avirulent T3SS-defective *hrcV* mutant CFBP13802 [1]. Leaf tissues were sampled 24 or 48 hpi. For each treatment and timepoint, 6 leaf discs from independent plants were pooled for total RNA extraction. After mRNA enrichment, RNA sequencing generated around 24M paired-end reads of 100bp per sample. Reads were filtered to remove adapters and low-quality reads. Salmon was used to map reads against the *P. vulgaris* reference genome and estimate the number of mapped reads (count.txt) [2]. Raw counts were normalised using EdgeR's *calcNormFactors* function from AskoR [3].
Data source location	*Institution: Growth chambers from Angers Plant Phenotyping Facility PHENOTIC, Institut de Recherche en Horticulture et Semences, UMR 1345 IRHS INRAE* *City: Beaucouzé* *Country: France*
Data accessibility	Repository name: Gene Expression Omnibus Data identification number: GSE271236 Direct URL to data: https://www.ncbi.nlm.nih.gov/geo/query/acc.cgi?acc=GSE271236 Instructions for accessing these data: Go to https://www.ncbi.nlm.nih.gov/geo/query/acc.cgi?acc=GSE271236
Related research article	

## 1 Value of the Data


 

• This dataset contains the transcriptome of bean leaves responding to the inoculation of either the wt virulent strain CFBP4885 of *Xcf* or its avirulent T3SS-defective *hrcV* mutant CFBP13802, or mock (water). The transcriptomic analysis was performed at 24 or 48 hpi.
• These data are a useful resource for the plant-microbe interactions community. Likewise, researchers interested in breeding for bean resistance to Common Bacterial Blight may find interesting molecular markers.
• This dataset may be used to address the underlying mechanisms involved in resistance to CBB, as the response of susceptible and resistant bean genotypes were compared.
• This dataset may be used to gain insights into the transition between pattern-triggered immunity (PTI) and effector-triggered susceptibility (ETS), as response of bean cultivars was monitored at 24hpi and 48hpi.

## 2 Background

Common bacterial blight (CBB), one of the most damaging diseases of bean, is characterized by greasy spots on aerial organs of plants, which develop into large necroses. Two different species of *Xanthomonas* can cause CBB, *i.e.* strains of *Xanthomonas citri* pv. *fuscans* (*Xcf*) and *Xanthomonas phaseoli* pv. *phaseoli* (*Xpp*) [4]. Despite variations in resistance among bean accessions, no major resistance gene has been identified [5,6].

A previous comparison of the transcriptomic response to *Xpp* of susceptible and resistant American bean cultivars highlighted mechanisms underlying resistance to CBB [6]. However, it remains unknown whether the same mechanisms in bean plants are involved in response to strains of *Xpp* and *Xcf,* or whether mechanisms underlying resistance in American and European cultivars are similar. Therefore, our dataset documents the response of European bean cultivars susceptible or resistant to *Xcf*.

The pathogenicity of *Xanthomonas* strains relies on the T3SS to inject T3Es into the plant cell. Mutation of *hrcV* abolishes the secretion through the T3SS and pathogenicity on bean [1]. To investigate the plant functions subverted by the injection of the whole set of T3Es by *Xcf*, our dataset documents the response of beans to the strain CFBP4885 and its T3SS-defective *hrcV* mutant CFBP13802.

## 3 Data Description

**Fig. 1:**
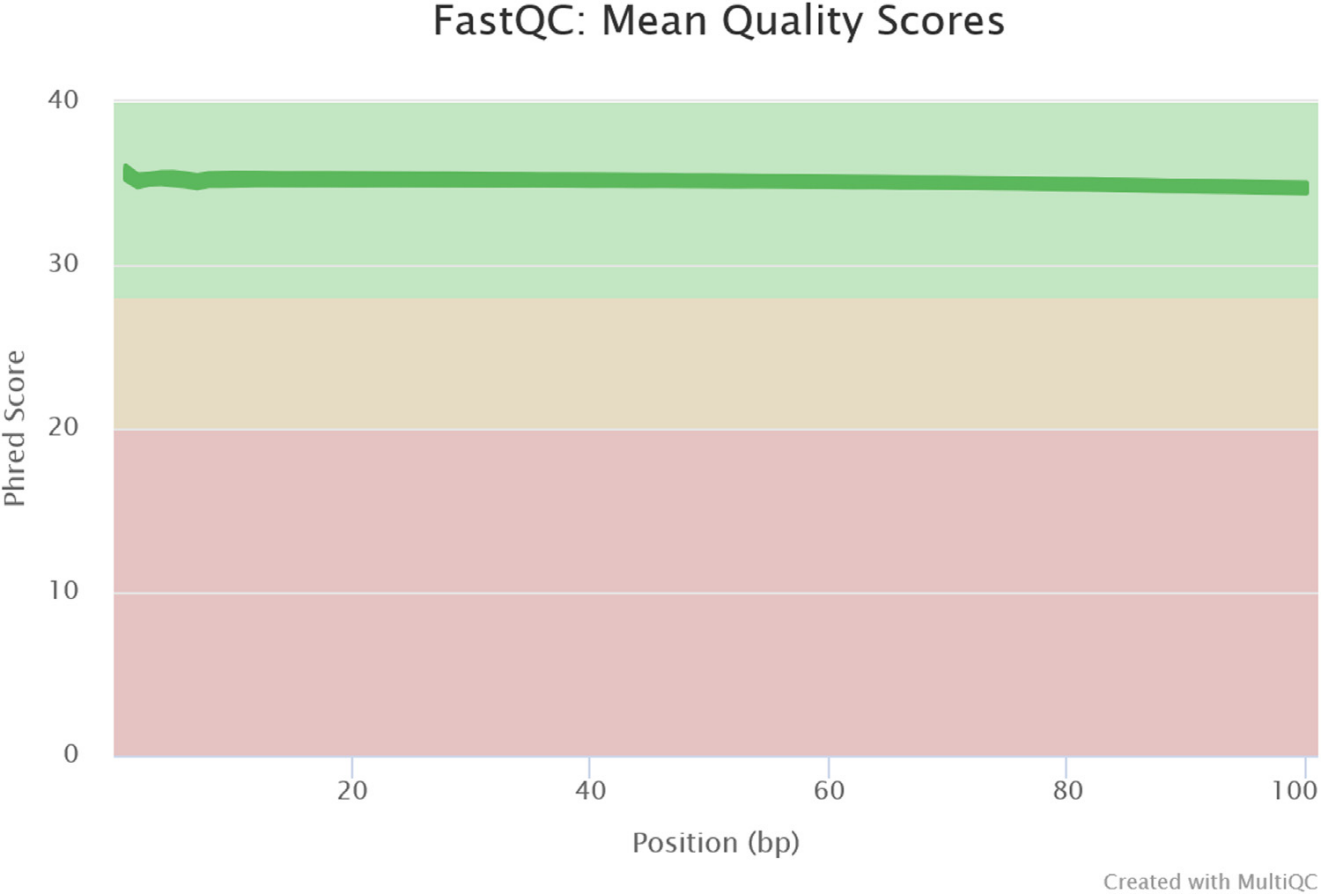
Overview of the range of quality values across all bases at each position in the fastq obtained from FastQC.

**Fig. 2:**
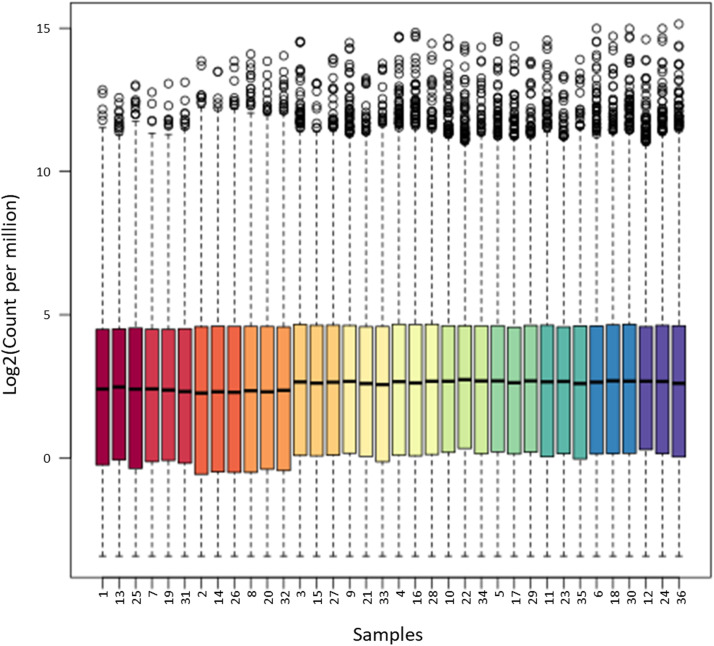
Boxplot distribution of the TMM normalized counts per sample.

**Fig. 3:**
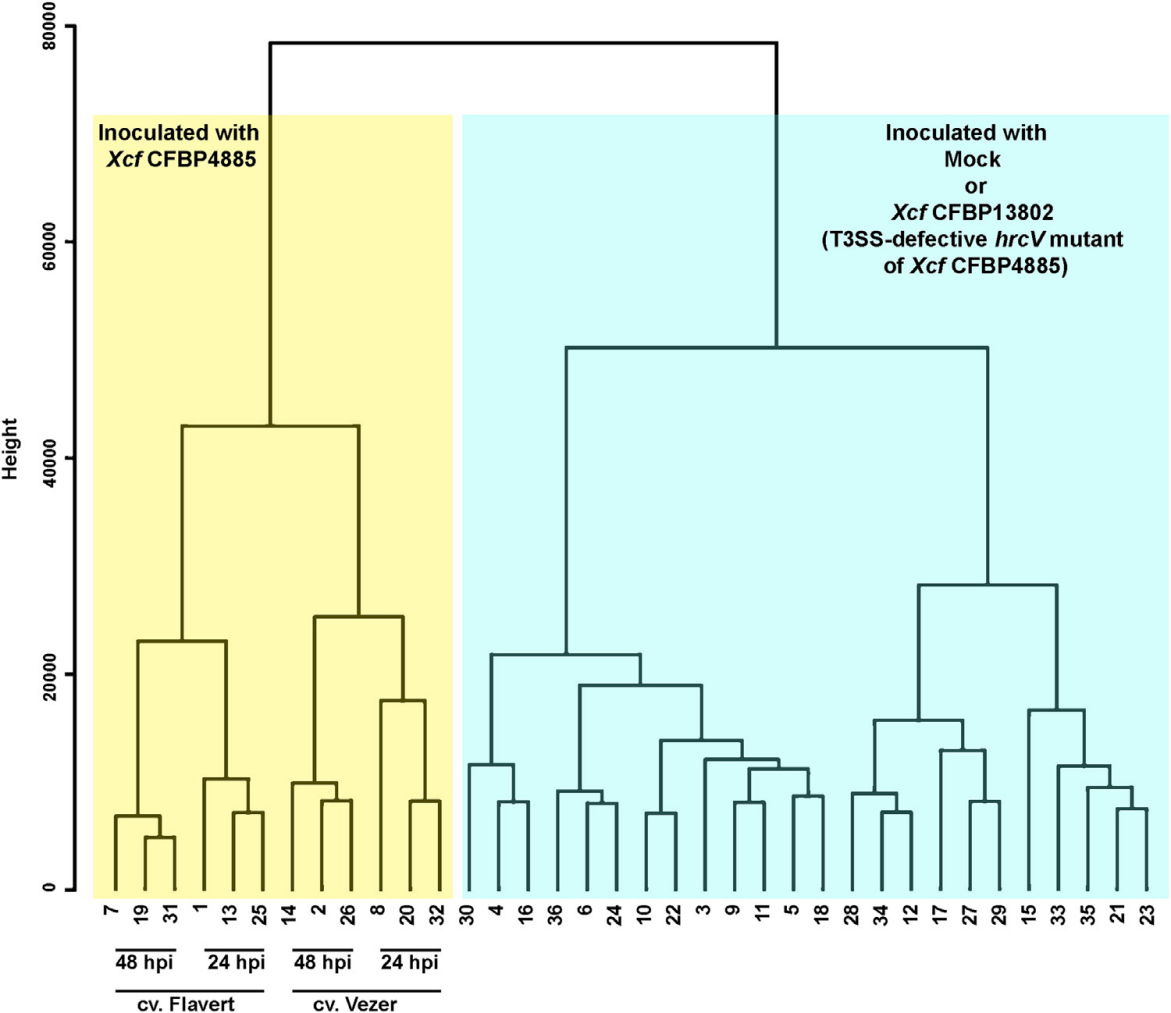
Hierarchical clustering analysis based on dissimilarity scores obtained from the TPM values to validate reproducibility of replicates. Samples are numbered from 1 to 36 according to Table 1. Samples divide into 2 distinct groups: i) samples inoculated with Mock or the *Xcf* avirulent *hrcV* mutant (CFBP13802) and ii) samples inoculated with the *Xcf* virulent strain CFBP4885. Among samples inoculated with *Xcf* CFBP4885, the hierarchical clustering analysis discriminates samples from the different cultivars. Finally, among samples of each cultivar inoculated with *Xcf* CFBP4885, the hierarchical clustering analysis discriminates samples harvested at 24 hpi or at 48 hpi.

This manuscript presents a transcriptomic dataset obtained from leaves of two cultivars of *Phaseolus vulgaris,* susceptible (Flavert) and resistant (Vezer) to CBB. Leaves were inoculated with either a virulent strain or an avirulent strain of *Xanthomonas* or Mock. Samples were collected at 24 hpi and 48 hpi. Table 1 lists all sample names along with information regarding inoculated strains, inoculated cultivars, sampling times, the number of the biological repetition, read lengths, the percentage and number of reads mapped using Salmon, and the total number of reads returned by BGI after total RNA sequencing. Fig. 1 shows a merged sequence quality report for all samples obtained from FastQC using MultiQC [7,8]. All 36 samples displayed Phred quality scores around 35, corresponding to a base calling accuracy of 99.95%. The Salmon algorithm (version 1.2.1) was used to map and quantify raw reads to the *P. vulgaris* reference genome, and the corresponding count table is provided in Table S1*.* Counts were normalized using TMM method from *calcNormFactors* function of EdgeR library [3] (Table S2) and boxplot distribution of TMM normalized counts per samples were displayed in Fig. 2. Fig. 3 presents a hierarchical clustering analysis based on dissimilarity scores obtained from the TMM normalized count values of the 36 samples. Fig. S1 illustrates the sampling procedure.

**Table 1:** Summary of sample files with the corresponding inoculated strain, bean cultivars, the time of sampling, the biological replicate, the length of the reads, the percentage (% Aligned) and number of reads mapped (M aligned, in million) using Salmon (version 1.2.1), and the total number of reads (M seq, in million) returned by BGI after total RNA sequencing.

## 4 Experimental Design, Materials and Methods

1. Bacterial growth conditions:
  The virulent *Xanthomonas citri* pv. *fuscans* strain *Xcf* CFBP4885 (Rif^R^) and its avirulent T3SS-defective *hrcV* mutant (CFBP13802) (Rif^R^, Kan^R^) were obtained from the French Collection of Plant-associated Bacteria (CFBP, IRHS, Angers, France. DOI: https://doi.org/10.15454/E8XX-4Z18). Bacterial strains were cultured at 28°C on Tryptic Soy Agar medium (17.0 g.L^−1^ tryptone, 3.0 g.L^−1^ glucose, 5.0 g.L^−1^ NaCl, 5 g.L^−1^ K_2_HPO_4_ and 15 g.L^−1^ agar) supplemented with Rifampicin (100 µg.mL^-1^) and/or Kanamycin (50 µg.mL^-1^).
2. Plant growth conditions:
  For this study, two cultivars of *Phaseolus vulgaris* were selected: Flavert (susceptible) and Vezer (resistant). The plants were grown in a controlled growth chamber under LED light (280 µmol·m^-2^·s^-1^) with a photoperiod of 16 h days and 8 h night. The day temperature was set at 23°C, while the night temperature was set at 20°C. Plants were grown in Traysubstrat 75/25 (Klasmann-Deilmann, Germany) and were watered with standard water until the first trifoliate leaf emerged. Upon appearance of the first trifoliate leaf, fertilized water (N-P-K: 15-10-30; pH 6.5; ec 1300 S.m^-1^) was used. The relative humidity in the growth chamber was set at 80%. Plants were incubated at 90% relative humidity for 24 h prior to the inoculation. Following inoculation, the relative humidity was set back to 80%.
3. *Xanthomonas* inoculation and leaf tissue sampling:
  Inoculation was performed once the plants had fully developed the first trifoliate leaf and had begun developing the second trifoliate leaf, which was approximately 16 days after sowing.
  Bacterial strains were grown overnight at 28°C on Tryptic Soy Agar medium supplemented with the appropriate antibiotics. For the preparation of inocula, bacteria were then resuspended in sterile water to reach an OD_600_ of 0.1 (equivalent to 1.0 × 10^8^ cfu.mL^-1^). Negative control (Mock) consisted in sterile water only.
  *Xanthomonas* suspensions and Mock were then infiltrated into the first trifoliate leaf using a vacuum chamber. The pressure was set to -1.10^6^ Pa and was maintained for 1 min. The homogeneity of the infiltration in leaf tissues was checked visually. In total, 72 plants were inoculated: six plants were inoculated for each inoculation condition (Mock, CFBP4885, CFBP13802), for each timepoint (24hpi and 48hpi), and for each cultivar (Flavert and Vezer).
  Sampling was performed by collecting leaf discs with a diameter of 1 cm from six plants in each modality. The collected leaf discs were pooled to buffer the variability between the six plant replicates (Fig. S1).
  The whole experiment was carried out on three independent plant lots grown three weeks apart. Over the three independent repeats of the experiment, 36 samples were collected for total RNA extraction.
4. RNA isolation and sequencing:
  Total RNA was extracted from leaves collected after strain inoculation using the Macherey-Nagel NucleoSpin® RNA plus kit (Macherey Nagel, Düren, Germany). RNA quality was assessed with a NanoDrop spectrophotometer (Thermo Scientific™ NanoDrop™ One). Samples with acceptable quality (260/280 and 260/230 absorbance ratios of 2) were sent to the Beijing Genomics Institute (BGI, Hong Kong) for further quality assessment using a Fragment Analyzer (Agilent Technologies, Santa Clara, CA, USA). The RNA Integrity Number (RIN) of the 36 samples averaged 7.32, and the 28S/18S ratio averaged 1.8. An oligo-dT enrichment on mRNA with poly A tail was performed by BGI. Library preparation and sequencing were conducted on the BGISEQ-500 platform, generating an average of 24 million paired-end reads of 100 base pairs per sample.
5. RNA-seq data analyses:
  The quality of fastq files was controlled using FastQC [7]. Reads were filtered to remove adapters and low-quality reads. Reads were mapped onto the *Phaseolus vulgaris* reference genome (*Pvulgaris_442_v2.1*; available at https://phytozome-next.jgi.doe.gov/info/Pvulgaris_v2_1) and transcript abundances were quantified with Salmon algorithm (version 1.2.1) using the quasi-mapping mode and the ‘–validateMappings’, ‘–useVBOpt’ and ‘–seqBias’ options [2]. Raw counts were normalized using TMM (Trimmed Mean of M-values) method of *calcNormFactors* function of EdgeR library from AskoR for each sample [3]. Reproducibility of replicates was validated by a hierarchical cluster analysis based on dissimilarity scores obtained from the normalized raw count values of the 36 samples using the ‘*cpm*’, ‘*dis*’ and ‘*hclust*’ functions in R (version 4.1.2).

## Limitations

None.

## Ethics Statement

This work does not contain any studies with human or animal subjects.

## CRediT authorship contribution statement

**Christopher Gihaut:** Conceptualization, Investigation, Methodology, Visualization, Writing – original draft. **Chrystelle Brin:** Investigation. **Martial Briand:** Software. **Jérôme Verdier:** Resources, Writing – review & editing. **Matthieu Barret:** Funding acquisition, Resources, Writing – review & editing. **Thomas Roitsch:** Supervision, Writing – review & editing. **Tristan Boureau:** Funding acquisition, Supervision, Conceptualization, Writing – original draft, Writing – review & editing.

## Data Availability

Transcriptomic dataset of Phaseolus vulgaris leaves in response to the inoculation of pathogenic Xanthomonas citri pv. fuscans and its type 3 secretion system-defective mutant hrcV. (Original data) (Gene Expression Omnibus - GEO).

## Appendix Supplementary materials

Table S1. Count table of mapped reads from the 36 samples using Salmon algorithm (count.txt). Image, application 1

Table S2. TMM normalised count table of mapped reads from the 36 samples using Salmon algorithm (TPM.txt). Image, application 2

Fig. S1. Overview of the experimental design. Image, image 3
