# Supplementary figures and images for: Transcriptomic dataset of Phaseolus vulgaris leaves in response to the inoculation of pathogenic Xanthomonas citri pv. fuscans and its type III secretion system-defective mutant hrcV
Source: Data Brief. 2024 Sep 21;57:110938. doi: 10.1016/j.dib.2024.110938 (PMC11492091; doi:10.1016/j.dib.2024.110938)

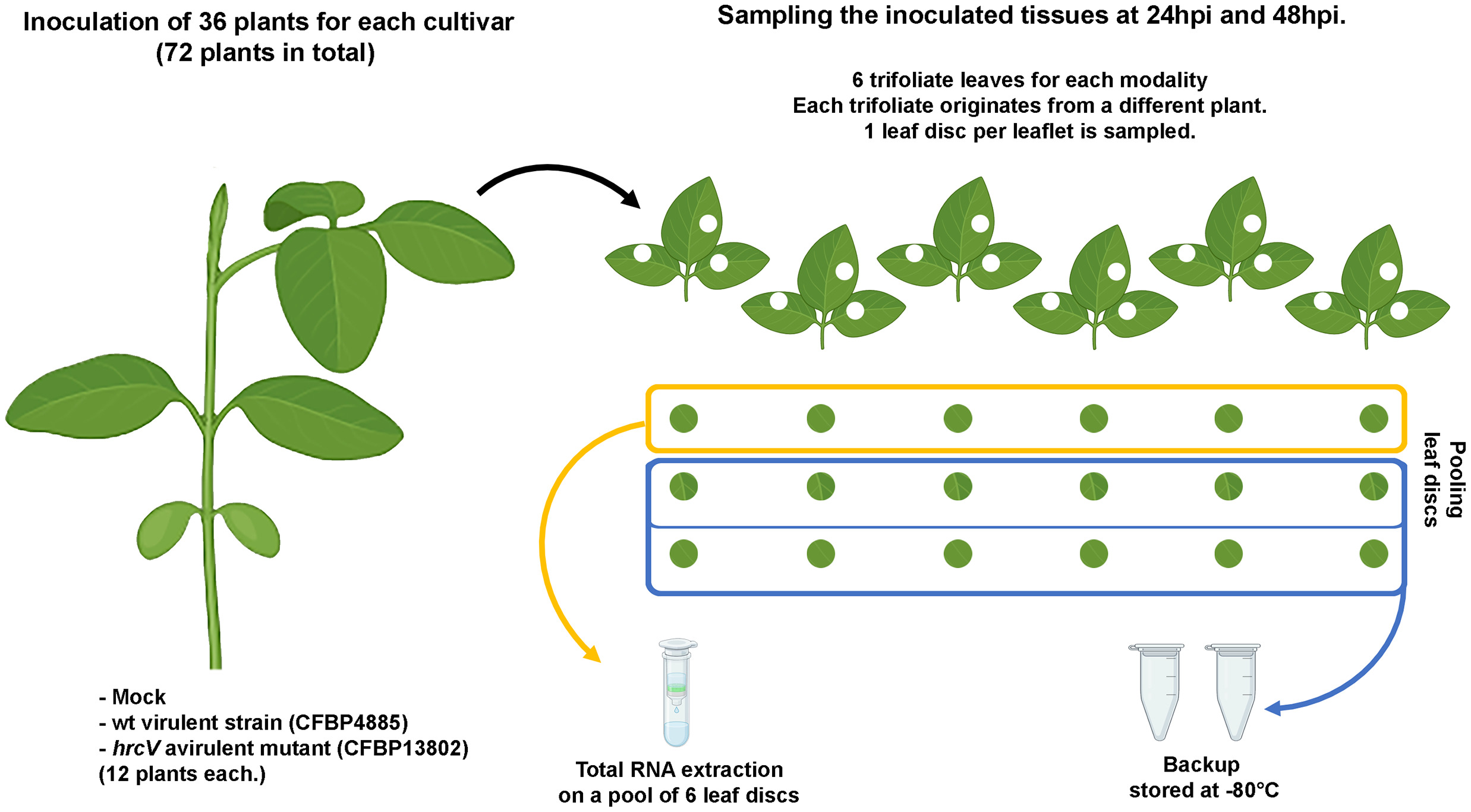

Supplement: Supplementary file 3 — Fig. S1. Overview of the experimental design. [file mmc3.jpg]
